# The Cistern—A Substitute for the Country Well

**Published:** 1897-04

**Authors:** Harvey B. Bashore

**Affiliations:** West Fairview, Pa.


### THE CISTERN—A SUBSTITUTE FOR THE COUNTRY WELL.

We have been preaching against the old well, but if we would make any progress in overcoming it, it is necessary that some substitute, which is easily available, inexpensive, and practical, should be presented. All this may be claimed for the cistern, if properly constructed and supplied with clean rain-water.

In the Atlantic States, at least, where the rainfall is about 37 inches annually, the supply is amply adequate and reliable. If the house roof is only 1,000 square feet—and many houses will furnish even more—the yearly yield of rain-water collectable will be about 20,000 gallons, which at ten gallons per head per day, is more than sufficient for the wants of an ordinary family.

The cistern, however, needs not be excessively large: One, ten feet in diameter and five feet deep, will hold 2,000 gallons; and this amount could last with ordinary use more than a month; but it is very rare indeed that a month passes without some precipitation. The construction of the cistern is of the utmost importance, for in its adaptability to keep out soil-water rests its superiority to the well. If made of brick and thoroughly cemented, it will be proof, in most cases, against contamination from soil-water. I have known such a cistern to last many years without a sign of seepage. In the next place we need a filter for rain-water; unless it is collected from a cleaner catch than is generally the case—from the roof—it has a peculiar flavor and odor, due to impurities washed off. For protection against these, a partition should be built in the cistern, from top to bottom. At the bottom there should be an opening connecting the two sides, or perhaps it would be a better way to build two cisterns side by side, and connect them with a small pipe.

In one apartment the filter should be constructed, three or four feet thick, with layers of sand, polarite, and gravel in the order named. The top layer of sand soon becomes covered with a film containing nitrifying bacteria which aids in making the filter very efficient. The rain-water leader from the roof should, of course, discharge into the filter-side, and the pump for drawing the water be confined to the other side.

With a cistern so made and connected, I can state positively that good, relatively pure, and wholesome drinking water may be obtained. Even the ordinary cistern, without a filter, if properly cared for, is vastly safer, and therefore more desirable than the old well. If the collection is made from a tin roof kept moderately clean, the water will be odorless, generally palatable, and harmless. Such drinking water the writer has commonly used without detriment.

The collecting surface, whether tin, wood or slate, should be kept as clean as possible, especially from the fall from overhanging trees. As an additional safeguard, it is a good plan to have a "cut off" in the roof-water leader, so that the first rainfall washing the roof may be turned off. The substitution of cisterns for wells generally would probably materially lessen the sickness rate of typhoid fevers and probably of some malarial diseases. The change should be urged by the sanitary authorities.

HARVEY B. BASHORE, M. D.

West Fairview, Pa., Dec. 9th, 1896.—*The Sanitarian.*

